# Reconstructing the History of Mesoamerican Populations through the Study of the Mitochondrial DNA Control Region

**DOI:** 10.1371/journal.pone.0044666

**Published:** 2012-09-19

**Authors:** Amaya Gorostiza, Víctor Acunha-Alonzo, Lucía Regalado-Liu, Sergio Tirado, Julio Granados, David Sámano, Héctor Rangel-Villalobos, Antonio González-Martín

**Affiliations:** 1 Department of Zoology and Physical Anthropology, Faculty of Biology, Complutense University of Madrid, Madrid, Spain; 2 Laboratorio de Identificación Genética, GENOMICA S.A.U. Grupo Zeltia, Madrid, Spain; 3 Laboratorio de Genética Molecular, Escuela Nacional de Antropología e Historia, Mexico City, Mexico; 4 División de Immunogenética, Departamento de Trasplantes, Instituto Nacional de Ciencias Médicas y Nutrición Salvador Zubiran, Mexico City, Mexico; 5 Academia de Cultura Científica – Humanística, Universidad Autónoma del Estado de México, Mexico City, Mexico; 6 Instituto de Investigación en Genética Molecular, Centro Universitario de la Ciénaga, Universidad de Guadalajara, Ocotlan, Mexico; University of Florence, Italy

## Abstract

The study of genetic information can reveal a reconstruction of human population’s history. We sequenced the entire mtDNA control region (positions 16.024 to 576 following Cambridge Reference Sequence, CRS) of 605 individuals from seven Mesoamerican indigenous groups and one Aridoamerican from the Greater Southwest previously defined, all of them in present Mexico. Samples were collected directly from the indigenous populations, the application of an individual survey made it possible to remove related or with other origins samples. Diversity indices and demographic estimates were calculated. Also AMOVAs were calculated according to different criteria. An MDS plot, based on FST distances, was also built. We carried out the construction of individual networks for the four Amerindian haplogroups detected. Finally, barrier software was applied to detect genetic boundaries among populations. The results suggest: a common origin of the indigenous groups; a small degree of European admixture; and inter-ethnic gene flow. The process of Mesoamerica’s human settlement took place quickly influenced by the region’s orography, which development of genetic and cultural differences facilitated. We find the existence of genetic structure is related to the region’s geography, rather than to cultural parameters, such as language. The human population gradually became fragmented, though they remained relatively isolated, and differentiated due to small population sizes and different survival strategies. Genetic differences were detected between Aridoamerica and Mesoamerica, which can be subdivided into “East”, “Center”, “West” and “Southeast”. The fragmentation process occurred mainly during the Mesoamerican Pre-Classic period, with the Otomí being one of the oldest groups. With an increased number of populations studied adding previously published data, there is no change in the conclusions, although significant genetic heterogeneity can be detected in Pima and Huichol groups. This result may be explained because populations historically assigned as belonging to the same group were, in fact, different indigenous populations.

## Introduction

The American continent was peopled across the Bering Strait from Asia after the LastGlacial Maximum (LGM) about 15,000 years before the present (YBP) [1]. Several models have been proposed to explain the human settlement of the continent. One of the most accepted theories [2] suggests that colonizing populations must have undergone a process of maturation and differentiation in Bering, prior to the settlement of the new world [3], [4], [5].

During the process of human expansion, fragmentation and diversification of the aboriginal populations increased. In fact, there are currently between 400 and 1,500 indigenous groups in America [6]. Mesoamerica is an area defined by cultural parameters [7] which extends from the northern part of today’s Mexico into Central America [8]. It is inhabited by 291 ethnic groups, among them are some of the most representative American cultures such as the Aztecs and the Mayas [6].

Different molecular genetic studies of Native American populations are based on the mitochondrial genome (mtDNA) and five haplogroups: A2, B2, C1, D1 and X2 [9], [10].

The characterization of a large number of Native Mexican populations should provide insights into the American model of colonization and expansion and the possible factors that have given rise to the current genetic and cultural diversity of the region.

In this paper 605 mtDNA control region sequences from eight indigenous Mesoamerican and Aridoamerican groups were analyzed. Note that Aridoamerica corresponds to the area proposed by Kroeber in 1939 [11] as “Greater Southwest”, and has been widely used in American literature [12]. Aridoamerica is a term used to describe a region of the southwestern United States and the northern and central regions of Mexico, in contrast to Mesoamerica which lies to the south and east. In this study it is represented by the Pima group.

## Results

### mtDNA Diversity and Demography in Mesoamerica

**Figure 1:**
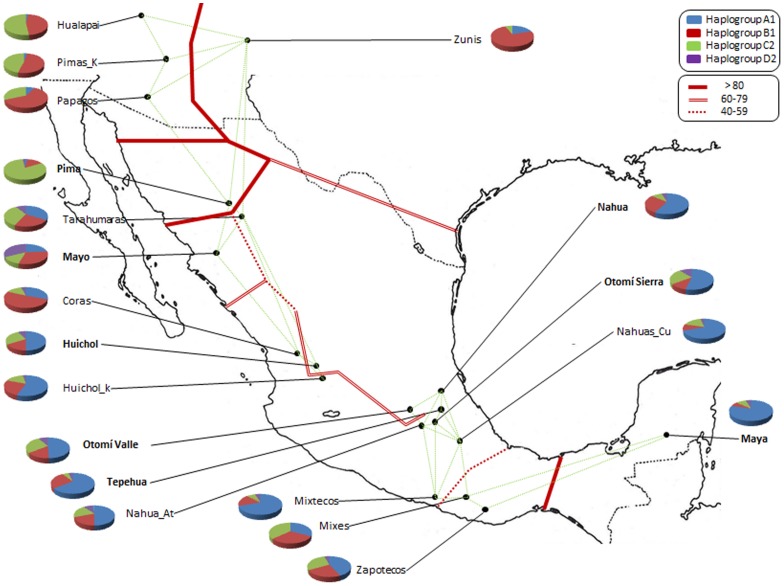
Populations analyzed. Geographical distribution of populations analyzed (in bold) and those used for comparison purposes. Pie charts show haplogroup frequencies. Red lines indicate genetic barrier intensity and green lines the Monmonier geometric traces used to construct the barriers.

The 605 samples (Table doc, supplementary information) came from 8 indigenous groups (Table S1, Table S2). Four major pan-American haplogroups (Figure 1) and five European haplogroups were detected analyzing the sequences between positions 16024 and 576 following CRS).

To determine whether the frequency distribution of haplogroups in Mesoamerica follows a geographical pattern, correlations between these and the variables latitude and longitude were calculated. In Mesoamerica, and hgC1 hgB2 show the Same trend, Increasing with latitude (r = 0.637, P = 0.03 and r = 0.465, = 0.039, for B and C respectivamente) and decreasing with longitude (r = −0,619, p = 0.004 for B and r = −0,517, P = 0.020 for C). While you have to reverse hgA2 trend, decreasing with latitude (r = −0,795, P<0.0001) and with Increasing longitude (r = 0.864, p<0.0001), presents no correlation hgD1 With The Above-Mentioned parameters.Genetic diversity indices are shown in Table 1 and, in general, are high: Ĥ = 0.99 and π = 0.0102. With respect to group diversity indices, two models can be described, even though variation is wide. The first group includes populations with high values (Otomí Sierra, Otomí Valle, Huichol and Nahua) whilst values in the second group are low (Pima, Mayo, Maya and Tepehua).

**Table 1:** Sequence diversity indices for mtDNA lineages in the control region (16024-576) in eight indigenous populations.

Population	N	K	S	Ĥ±sd	π±sd	θ	D
Pima	49	17	34	0.89±0.028	0.0053±0.0006	5.93	−0.74
Mayo	55	33	52	0.95±0.017	0.0096±0.0003	10.73	−0.18
Huichol	36	25	41	0.96±0.019	0.0096±0.0007	10.76	0.31
Nahua	189	92	106	0.98±0.003	0.0098±0.0003	10.75	−1.31
Otomí Valle	81	39	61	0.96±0.008	0.0097±0.0003	10.88	−0.37
Otomí Sierra	90	52	83	0.97±0.008	0.0103±0.0003	11.55	−0.96
Tepehua	51	25	37	0.94±0.017	0.0089±0.0005	9.03	0.36
Maya	44	25	49	0.95±0.018	0.0077±0.0009	8.30	−0.92
Total	600	269	177	0.99±0.001	0.0102±0.0001	10.72	−1.77*

* level of significance of 0.05.

N, sample size; K, number of different sequences; S, number of polymorphic sites; Ĥ, sequence diversity; π, nucleotide diversity; θ, mean number of pairwise differences between sequences; D, Tajima test of selective neutrality (sd represents the standard deviation).

No significant values of Tajima’s D were found in the study groups. All eight populations therefore fit the neutral theory model. This result contrasts with the value obtained for all populations taken together (D = −1.77, p<0.005), in which there is an excess of rare haplotypes, indicating a rapid growth of the indigenous population.

θπ, θ_K_ and θ_S_ indices (Table 2) are population estimators expressing female effective population size. Recent female population sizes are lower in Pima and Tepehua populations, while higher sizes are found in Otomí Sierra and Nahua. Regarding θπ, the Pima population has lower values (8.78±4.57) than the others, whose values range between 11.14±5.73 for Mayas and 14.47±7.24 for Otomí Sierra.

**Table 2:** Theta estimators of eight indigenous populations from Mesoamerica and Aridoamerica.

Population	θ_K_ (95% CI)	θ_S_± sd	θ_π_± sd	
Pima	8.80 (4.80–15.80)	7.62±2.44	8.78±4.57	
Mayo	33.92 (19.98–57.93)	11.58±3.47	14.26±7.20	
Huichol	34.94 (18.04–69.32)	9.89±3.25	13.18±6.75	
Nahua	67.48 (50.20–90.56)	18.71±4.38	13.51±6.75	
Otomí Valle	28.94 (18.55–45.00)	12.28±3.42		13.76±6.92
Otomí Sierra	44.76 (29.49–67.98)	16.33±4.35		14.47±7.24
Tepehua	17.87 (10.38–30.61)	8.37±2.61		11.42±5.84
Maya	23.19 (12.92–41.75)	11.72**±**3.65		11.14**±**5.73

### Genetic Structure of Indigenous Populations

Two matrices representing genetic distances (F_ST_) and geographical distances were calculated. Correlation between these matrices was checked using the Mantel test [13] and the results suggested no correlation between genetic and geographic distances (r = 0.352, p = 0.160).

AMOVAs were also performed to detect population structure according to geographical, cultural, and linguistic criteria (Table 3). Based on geographical criteria, classification III was significant (F_CT_ = 0.0953; p = 0.0371). In this group, and unlike other geographical associations, it was decided that western slope populations in Sierra Madre Occidental, such as both Otomi groups and the Nahuas de la Huasteca, should be included in the Mexican central highland group, while those that were located on the eastern side, such as the Tepehuas, were classified as coastal. A detailed analysis of this geographic association shows that it overlaps with cultural regions [14].On the other hand, no significant values were found for linguistic groups, indigenous regions, or for the history of indigenous groups.

**Table 3:** AMOVAS based on different classification criteria.

Grouping criteria	Groups	Populations		Variance	Fixation índices	P
Geography I	North	Pima, Mayo	Within populations	84,13	Fst = 0,1586	0,0000*
	South	Maya	Among populations within groups	7,03	Fsc = 0,0771	0,0000*
	Center	Huichol, Nahua, OtomíValle, OtomíSierra, Tepehua	Among groups	8,83	Fct = 0,0883	0,0948
Geography II	North	Pima	Within populations	86.36	Fst = 0.1363	0.0000*
	West	Mayo, Huichol	Among populations within groups	4.73	Fsc = 0.0519	0.0000*
	Center	Otomí Valle	Among groups	8.90	Fct = 0.0890	0.0664
	Coast	Otomí Sierra, Tepehua,Nahua				
	Southeast	Maya				
Geography III	North	Pima	Within populations	85.79	Fst = 0.1421	0.0000*
	West	Mayo, Huichol	Among populations within groups	4.67	Fsc = 0.0516	0.0000*
	Center	Otomí Valle, Otomí Sierra, Nahua	Among groups	9.54	Fct = 0.0953	0.0371*
	Coast	Tepehua				
	Southeast	Maya				
Languages	Yuto-Nahua	Pima, Huichol, Mayo,Nahua	Within populations	89.37	Fst = 0.1062	0.0000*
	Otomangue	Otomí Sierra, Otomí Valle	Among populations within groups	15.60	Fsc = 0.1486	0.0000*
	Totonaco-Tepehua	Tepehua	Among groups	−4.98	Fct = −0.0497	0.5904
	Maya	Maya				
Indigenousregions	Norte	Pima	Within populations	86.27	Fst = 0.1373	0.0000*
	Mayo	Mayo	Among populations within groups	3.78	Fsc = 0.0419	0.0000*
	Huicot	Huichol	Among groups	9.95	Fct = 0.0995	0.1006
	Huasteca	Otomí Sierra, Nahua,Tepehua				
	Otomí	Otomí Valle				
	Yucatán	Maya				
History	Chichimecas	Pima, Mayo	Within populations	87.84	Fst = 0.1215	0.0000*
	Aztecas	Nahua, Tepehua	Among populations within groups	9.39	Fsc = 0.0965	0.0000*
	Tarascos	Huichol	Among groups	2.77	Fct = 0.0277	0.2746
	Otomí	Otomí Valle, OtomíSierra				
	Mayas	Maya				

* Significance level 0.05.

There are a number of distinct regions within Mesoamerica that are defined by a convergence of geographic and cultural attributes, being more conceptual than culturally meaningful. The demarcation of their limits is not rigid. The areas include Maya, Central Mexico, West Mexico, the Gulf Coast Lowlands, Oaxaca, the Southern Pacific Lowlands, and Southeast Mesoamerica (including northern Honduras).

### History and Origin of the Indigenous Populations

**Figure 2:**
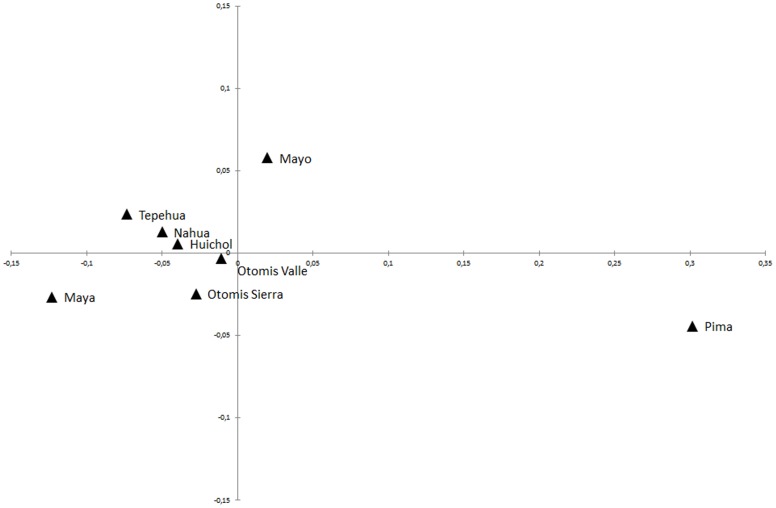
Multidimensional scaling based on the F_ST_ distances of eight indigenous populations from Mesoamerica and Aridoamerica (Stress = 0.097).

F_ST_ distances represented in a MDS plot (stress = 0.097) (Figure 2) showed the genetic relationships among the eight indigenous groups. There is a cluster of central and southern highland populations, which shows Maya differentiation, with Nahua and Tepehua near them, and an expected proximity between Otomí groups. Distributed at a considerable distance from this group are Mayo and Pima.

The four median-joining networks (Figures S1, S2, S3 and S4) reflect the unique haplogroups shared by all eight populations. All of them have a star-like model, typical of modern populations which have undergone rapid demographic growth.

The A2 median-joining network (Figure S1) has two central haplotypes, characterized by the presence/absence of C16111T mutation, dated to 3960.72 YBP. The oldest haplotype is represented by all groups except the Pima and Mayo. The most common derived haplotype has the same population composition, although the Maya population is not included. The B2 network (Figure S2) shows a clear central node consisting of five populations, all except Pima, Maya and Tepehua. The A16182C polymorphism (4064.55 YBP) characterizes the most common secondary node formed by the northern populations and Nahua. From the central node we can notice a derived haplotype defined by the C16295T variant (4606 YBP), which is observed in Otomí Sierra, Nahua and Tepehua. The central haplotype in C1 network (Figure S3) includes all populations except Huichol. Another interesting point is the presence of a node consisting of Pima and Mayo unique haplotypes (polymorphism C16295T dated to 2984.49 YBP). The D1 network (Figure S4) shows a central node that contains five groups. In this node there is a branch exclusively represented by both Otomí populations and characterized by the presence of the G16274A polymorphism (4145.85 YBP).

### Genetic Relationships and History of Populations

In order to obtain a broader view of the diversity and history of Mesoamerica, we compared data for twelve indigenous populations from the scientific literature [15] (Figure 1).

Note that in this paper the terminology used by the National Commission for the Development of Indigenous Peoples (CNI Mexico) has been followed. For this reason groups O’odham Akimel O’odham and Tohoma have been called Pimas_k and Papago. The diversity and demographics indices of these populations are presented in Table S3 and Table S4. In the MDS (Figure S5), the northern populations can be clearly distinguished from those in Mesoamerica. Figure S5 has been done from the Fst calculated between the 20 populations (Table S6). This representation has been built AMOVAs were calculated for these populations using different classification criteria (Table S5). Geographical groupings are significant, but again language groups are not significant. Examination of the shared haplotype distribution (Table S7) provides useful information about the phylogenetic relationships and the origin of the different groups.

The geographic discontinuities in the haplogroup frequency were computed, detecting genetic boundaries that delimit geographical regions (Figure 1). The most important genetic boundaries (higher than 80%) were detected in the western region between Aridoamerica and the central highlands. Here, it is interesting to note the behavior of the Pima population. Other barriers with values between 60 and 79% mark the Mesoamerican longitudinal separation, differentiating the western highlands from central and eastern regions, although this becomes diluted in more southern regions. Oaxaca populations remained relatively isolated from the center by two barriers of unequal intensity.

## Discussion

The aim of this study is provide information about the origin, phylogenetic relationships, and history of Mesoamerican indigenous groups. Samples representing the different cultural and geographical areas of Mesoamerica and Aridoamerica were collected by the investigators directly from indigenous communities. This strategy permitted the exclusion of relatives and samples of dubious origin, which guaranteed they were representative of the selected indigenous groups. The analysis of 605 complete mtDNA control region sequences for seven Mesoamerican and one Aridoamerican indigenous groups makes it possible to reconstruct the female biological past of these populations.

The four Pan-American haplogroups arrived on the continent at the same time [16], and their distribution reflects the original pattern of settlement of the Americas and the subsequent genetic differentiation of Native American populations within these continental areas [17]. The majority of mtDNA sequences obtained belong to the four American haplogroups, although five haplogroups of European origin were identified. The fact that all of the people included in the study speak an indigenous language suggests that individuals of European maternal origin were integrated into the community generations ago. These European haplogroups were found in populations of the Sierra Madre Oriental, which supports the idea that the permeability of this mountain ridge served as a natural pass between coastal and highland areas.

The distribution of Amerindian haplogroups does not fit the general model proposed by some authors: hgA2 north-south gradient, hgC1 and hgD1 south-north gradients, and a lack of distribution for hgB2 [18], [19].These results suggest that the general continental trend is diluted when performing more detailed geographical studies. On the other hand, no hgX2 haplogroup was detected because its distribution is confined to northernmost areas [20], [21].The pattern of mtDNA diversity in Native American populations should explain the distribution of these populations with the neutral genetic diversity model. This assumes a single founding population that grows in size and creates derived populations [22]. As the process continues, the newly-formed populations increasingly move away from the ancestral population, remaining relatively isolated while occupying new territories [23]. However, one consequence of this model is a largely hierarchical pattern of neutral genetic diversity and a linear decline in population genetic and phenotypic diversity with increasing geographical distance from the Bering strait [24]. In this case this principle was not found, and no correlation was detected (Table S8) between distance from the Bering Strait and our genetic diversity parameters. The explanation for this result may involve the demographic history of these populations, secondary movements, and gene flow that could mask this relationship [25].

Genetic diversity is an estimation of a population’s genetic variation and the result of its demographic history. In this sense, the lowest values were detected in Pima, Tepehua and Mayo populations, although each of them does so for different reasons. The first two populations were tipically hunter-gatherer communities until recently [26], [27], which implies a lower tendency for mobility, small population size and important genetic drift effects. The Tepehua are different because they are one of the smallest indigenous Mesoamerican groups [28]. On the other hand, among the populations with greater genetic diversity are both the Otomí groups and the Nahua, a logical result since they are the ones with the greatest presence in the region.

The demographic model based on genetic diversity is corroborated by the results of the θ and Tajima’s D indices. Groups with a high diversity index show negative values for D, suggesting an excess of rare alleles in the population and high values of θ_K_ and θ_S_, while populations with a lower diversity index usually present positive values for D and moderate values for θ_K_ and θ_S_.

Mesoamerican groups have a very similar demographic history characterized by ancestral demographic uniformity, and have grown over time. Northern populations differ from this model, however, since some are based on smaller sizes than the Mesoamerican populations, though they have grown in recent times.

AMOVA results verify that Mesoamerican populations are genetically structured according to geographical parameters. This structure, allows us to draw a distinction between a northern region and Mesoamerica, which in turn can be subdivided into “East”, “Center”, “West” and “Southeast”. It is noteworthy that this geographical description matches the cultural area classification. The fact that these two areas overlap would seem to indicate that a combination of geographical distribution and relative isolation was at the origin of the blossoming of these indigenous cultures. The language parameter was found not to influence the distribution of genetic markers [29].

The distribution of populations in the MDS plot (Figure 2) provides information on the genetic similarities between indigenous groups. In this case, there is a clear difference between central highland and northern populations. This information is supported by MDS plot using the twelve populations (Figure S5). On the one hand, the Maya group, the only southeast population, is relatively separated from the Mesoamerican groups, which could indicate a common origin and also a period of isolation from the highland populations. This topography may be the product of a common ancestry and/or result of recent gene flow process. In order to discern whether the MDS results are due to one of these possibilities, networks for the four haplogroups were built.

The four networks feature a star pattern, indicative of populations that have experienced processes of explosive growth. In some of the four central nodes of the four haplogroups, eight populations are represented, which confirms single, unique origin for all of theml. A global analysis of the network information enables us to propose a general diversification model in Mesoamerican indigenous populations. The node defined by variant C16295T in B2 network, represented by Nahua, Otomí Valle and Tepehua, in addition to the absence of haplotypes belonging to Otomí Valle suggests that this node may be a product of admixture in the Sierra Madre Oriental. This information is also confirmed by the high number of derived haplotypes shared by the Sierra Madre populations. The G16274A variation in D1 network (4145.85 YBP) defines an exclusively Otomí node, the first ethnic group differentiated from the common gene pool. The presence of a unique Pima and Mayo haplotype dated to 2984.49 YBP (T16295C polymorphism of C1) which does not include the Nahua people and the polymorphism A16182C (4064.55 YBP) in B2 marks the possible time range for the separation of the precursors of the Aztec culture, that is to say the Nahua, and the northern groups. The two central nodes in A2 are composed of the same groups with one exception: the Maya population found in the principal node but not in derivatives. This gives scope to the suggestion that when the C16111T variant appeared, separating both nodes (dating to 3960.72 YBP), the Mayan group was already differentiated from the rest of the population pool. The network information is not detailed enough to provide information on the history of the Huichol and Tepehua groups. In fact, virtually no haplotypes of these populations appear outside the central node, which would indicate they are of a relatively recent origin. Only a few B2 variants typical in Tepehua date the origin of this population to the Middle Classic period 3071 YBP.

The barrier software result is consistent with the descriptive information about haplogroup distribution. The clearest genetic boundaries were detected in the Pima population, although this frontier extends northwards, separating the Zuni from other northern populations. Another deep limit is detected in the southern region, isolating the Maya from the remaining Mesoamerican populations. The next frontier in intensity divides the Mesoamerican region into two strands, the Atlantic and Pacific regions. Finally, note that lowest-intensity genetic boundaries were detected in Oaxaca populations relative to other central highland populations located in the Center of the Mexican territory.

The case of the Pima indigenous group is interesting since some results (MDS and barriers) show them to be genetically distinct. There are several possible explanations for these differences. One is that they may be the result of a sampling problem. A more plausible option, however, is that the Pima group is more heterogeneous than had previously been considered. This heterogeneity could be due to admixing with other groups in a post-colonization period, as has been detected in other groups in the New Continent [24], or it could be an older phenomenon. Ethnographic studies uphold the hypothesis that in the pre-Columbian period Pima were composed of two culturally distinct groups distributed in the north and south of the region currently occupied [28]. In the Huichol group the same phenomenon is detected and, in fact, some authors point to the existence of a dual origin for these populations and, therefore, the existence of an internal substructure that would justify the differences detected between Huichol people and huichol_k.

### Conclusions

Although it was not the objective of this study, the data point to a late colonization of the continent by Asian populations, and a shared, single origin for all of them. The first groups that came to the Mexican highlands colonized the region in a process of expansion and rapid growth in accordance with a model of settlement, expansion and differentiation. This process of fragmentation or tribalisation occurred mainly on the basis of geographical parameters but in the process of expansion did not necessarily follow a north-south axis [30].

Geographical isolation was not absolute, however, since gene flow has been detected in pre-Hispanic periods [31]. Nevertheless, isolation was sufficient to generate the existence of a clear genetic structure. This result confirms that geography acts as the main driving force in shaping the pattern of genetic variation that we observe today.

The differences of demographic histories between Aridoamerica and Mesoamerica are the result of very different survival strategies; in the north a hunter-gatherer strategy and in the center and southeast a sedentary agriculture, with livestock, trade and sometimes complex societies.

The Otomí population was the first of these populations to separate from the original gene pool and supports the hypothesis that the Otomí culture is one of the oldest in Mesoamerica [32]. The next segregation is detected between the northern populations and the Nahua who have strong ties with the northern groups [33], [34]. This differentiation occurred in the late Early Preclassic (2500-1200 YBP) or, at the latest, during the Middle Preclassic (1200-400 YBP). The Maya differentiated from the central highland populations nearly 4,000 YBP in the Mesoamerican Preclassic (2,500-1,200 YBP). Interestingly, the dates of coalescence correspond to the archaeological information [35]. No clear information has been obtained to allow us to put forward a hypothesis concerning the history and origin of the Huichol and Tepehuas groups, although the data would support the idea of a more recent origin, probably during the middle Classic.

Gene flow between population and admixture with European people was detected in the region of the Sierra Madre Oriental. This result shows that orographically complex regions can be permeable to gene flow as long as certain precise circumstances exist, in this case a natural passageway between two regions. Anyway it should be remembered that the mtDNA only provides information about femenine history and that in America, male and female migrations were different, as shown by studies based on Y chromosome markers [15], [36], [37].

We have detected a high intra-population heterogeneity in Pima and Huichol groups. This result shows that the genetic diversity of the indigenous population in pre-Hispanic times was greater than currently. It is also possible that many indigenous groups were considered and classified as the same group when they were in fact different groups sharing the same geographical area. On the other hand, the heterogeneity of the Nahuas supports the idea that this group is composed of genetically distinct cultural groups absorbed by the Aztecs.

Apart from the well-known limitations of mtDNA in reconstructing the history of our species, we consider it an appropriate tool with which to address the reconstruction of the history of mankind. The study of the mtDNA control region and detailed and thorough sampling are powerful tools for investigating our past.

## Materials and Methods

### Sample Collection and Ethical Statements

In order to gain an accurate estimate of genetic variability, a careful selection of the indigenous communities was undertaken (Table S1). Each participant was told about the objective of the project and signed a consent document following the Helsinki protocol for the use of biological samples. An ethics approval statement was obtained for this study from the research and ethics committee of Hidalgo Autonomous University (Mexico).

Participants completed a survey to collect data relating to their origins and predecessors. Only those people who spoke an indigenous language were included in the study, with all four grandparents belonging to a particular group and coming from the selected region.

### Mitochondrial DNA Amplification and Sequencing

The D-loop or control region was sequenced for all 605 individuals using the Applied Biosystems BigDye Terminator kit (v1.1), in two or three fragments depending on the samples between positions (16024 and 576 following CRS).Amplified fragments were analyzed on Applied Biosystems genetic analyzers, ABI PRISM 3100 Genetic Analyzer and ABI PRISM 3130. All sequences are available from Table S9.

### Statistical Analyses

Various statistical analyses focused on the 600 control region mtDNA sequences of the eight indigenous groups were performed. The results were then compared with those available in the scientific literature [19].

A first descriptive approach involved the allocation of haplogroups [38] using various online resources: MitoMap [39]; MitoSearch (http://www.mitosearch.org); PhyloThree [40]; mtDB [41] and MitoTool [42]. From this classification, networks were constructed for each of the haplogroups using the program Network 4.6.0 [43]. Next, the same software was used to calculate coalescence times on the basis of a mutation rate of one change every 9,213 years. This calculation was obtained by analyzing the mtDNA of Alaskan human remains from 10,300 YBP [15].

Subsequently, the sequences were aligned and edited when necessary with the Bioedit 7.0.5 sequence alignment editor [44]. The following estimators were calculated: number of different sequences (K); number of polymorphic sites (S); gene diversity (Ĥ) and standard error; nucleotide diversity (π) and the number of differences between two sequences (θ). The female effective-population sizes were assessed by the computation of the estimators θπ, θ_K_ and θ_S_ [45]. The last two are recent size reporters but θ_K_ expresses better the recent female population effective size [46]. θπ is more sensitive to older demographic events. F_ST_ distances [47] and AMOVAs (Analysis of Molecular Variance) using different grouping criteria were also calculated. A Tajima’s D index was also calculated [48] to determine whether the population is evolving under the neutral theory model. All these parameters were calculated using the Arlequin 3.5 [49] and DnaSP [50] software. From F_ST_ distances, and using XL-STAT 3.2 software, an MDS (Multidimensional Scaling) was plotted in order to establish approximate relationships among populations. The Monmonier’s maximum difference algorithm [51] using Barrier v2.2 software [52] was applied to identify putative boundaries. This method has been widely used for the detection of genetic barriers between populations [53], [54], [55], [56].

## Supporting Information

Figure S1 **Median-joning network of the mtDNA sequences belonging to the A2 haplogroup in the indigenous populatons.** (numbers represent the position of mutation defining a haplotype).
(TIF)

Figure S2 **Median-joning network of the mtDNA sequences belonging to the B2 haplogroup in the indigenous populatons**. (numbers represent the position of mutation defining a haplotype).
(TIFF)

Figure S3 **Median-joning network of the mtDNA sequences belonging to the C1 haplogroup in the indigenous populatons**. (numbers represent the position of mutation defining a haplotype).
(TIF)

Figure S4 **Median-joning network of the mtDNA sequences belonging to the D1 haplogroup in the indigenous populatons**. (numbers represent the position of mutation defining a haplotype).
(TIF)

Figure S5 **Multidimensional scaling (MDS) based on F_ST_ distances of twenty populations from Mesoamerica and Aridoamerica (Stress = 0.145).**
(TIF)

Table S1 **Sample geographic and demographic information.** The percentage of indigenous population has been calculated in reference to the municipality (INEGI 2000).
(DOCX)

Table S2 **Number of samples (N) and haplogroups found in the eight populations.**
(DOCX)

Table S3 **Diversity indices for mtDNA control region (16024-576) sequences and lineages in twelve indigenous populations** [**15**] **.** N, sample size; K, number of different sequences; S, number of polymorphic sites; Ĥ, sequence diversity; π, nucleotide diversity; θ, mean number of pairwise differences between sequences; D, Tajima test of selective neutrality (s.d. standard deviation).
(DOCX)

Table S4 **Theta estimators for the twelve populations** [**15**] **.**
(DOCX)

Table S5 **AMOVAs based on different classification criteria.** We have used the mtDNA control region of twenty populations from Mesoamerica and Aridoamerica. In bold the eight populations studied in this paper. *Significance level 0.05.
(DOC)

Table S6 **F_ST_ calculated for twenty populations from Mesoamerica and Aridoamerica.** In bold the eight populations studied.
(DOC)

Table S7 **Number of haplotypes shared between the twenty populations.** Lower hemimatrix shows the number of haplotypes shared in the control region, upper hemimatrix shows the number of haplotypes shared in HV-I region. In bold the eight populations studied in this paper.
(DOC)

Table S8 **Pearson correlation coefficients (r) and their significance (p) between geographic distance from Bering Strait to the communities and genetic diversity indices.** K, number of different sequences; S, number of polymorphic sites; Ĥ, sequence diversity; π, nucleotide diversity; θ, mean number of pairwise differences between sequences.
(DOC)

Table S9 **Control region haplotypes distribution in the eight populations studied.**
(XLSX)
