# Supplementary figures and images for: Reconstructing the History of Mesoamerican Populations through the Study of the Mitochondrial DNA Control Region
Source: PLoS One. 2012 Sep 19;7(9):e44666. doi: 10.1371/journal.pone.0044666 (PMC3446984; doi:10.1371/journal.pone.0044666)

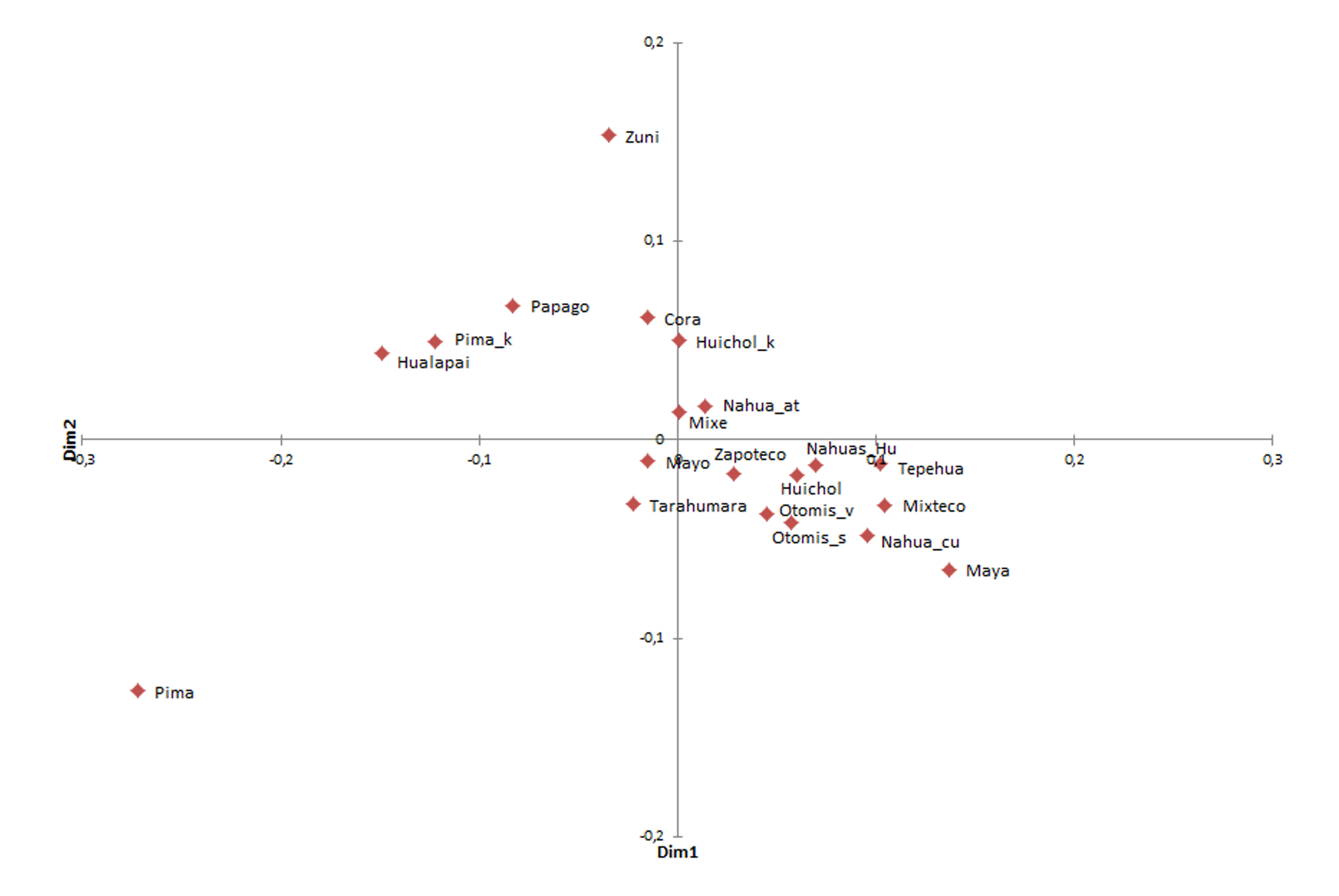

Supplement: Figure S5 — Multidimensional scaling (MDS) based on FST distances of twenty populations from Mesoamerica and Aridoamerica (Stress = 0.145). (TIF) [file pone.0044666.s005.tif]
