# Supplementary material for: Reconstructing the History of Mesoamerican Populations through the Study of the Mitochondrial DNA Control Region
Source: PLoS One. 2012 Sep 19;7(9):e44666. doi: 10.1371/journal.pone.0044666 (PMC3446984; doi:10.1371/journal.pone.0044666)
Supplement: Table S1 — Sample geographic and demographic information. The percentage of indigenous population has been calculated in reference to the municipality (INEGI 2000). (DOCX) [file pone.0044666.s006.docx]

| **Group** | **State** | **Municipality** | **Community** | **Latitude** | **Longitude** | **% Indigenous population** | **Indigenous**  **language** |
| --- | --- | --- | --- | --- | --- | --- | --- |
| Pima | Chihuahua | Temosáchi | Yepachi | 28º 25’ 19’’ | -108º 22’ 39’’ | 17,76 | Pima |
|  |  |  | Piedras Azules | 28º 24’ 60’’ | -108º 18’ 59’’ | 10,52 | Pima |
|  | Sonora | Yécora | Yécora | 28º 22’ 16’’ | -108º 55’ 32’’ | 4,45 | Pima |
|  |  |  | Juan Diego de los Pimas | 28º 21’ 57’’ | -108º 55’ 01’’ | 9,50 | Pima |
|  |  |  | El Kipur | 28º 24’ 19’’ | -108º 35’ 45’’ | 21,66 | Pima |
| Mayo | Sinaloa | El Fuerte | Los Capomos | 26º 25’ 32’’ | -108º 30’ 57’’ | 5,76 | Mayo |
| Huichol | Jalisco | Mezquitic | San Sebastián de Teponahuastlán | 22º 04’ 51’’ | -104º 03’ 49’’ | 1,47 | Huichol |
| Tepehua | Hidalgo | Huehuetla | Huehuetla | 20º 27’ 33’’ | -98º 04’ 35’’ | 58,22 | Tepehua |
| Nahua | Hidalgo | Huejutla de Reyes | Santa Catarina | 21º 05’ 56’’ | -98º 22’ 48’’ | 1,17 | Nahualt |
|  |  | Atlapexco | Pahactla | 20º 58’ 28’’ | -98º 21’ 31’’ | 7,44 | Nahualt |
|  |  |  | Tecacahuaco | 20º 56’ 29’’ | -98º 20’ 21’’ | 9,20 | Nahualt |
|  |  | Yahualica | Tlalchiyahualica | 20º 58’ 13’’ | -98º 23’ 59’’ | 11,24 | Nahualt |
|  |  |  | Atlalco | 20º 54’ 53’’ | -98º 19’ 41’’ | 3,52 | Nahualt |
|  |  | Xochiatipán | Texoloc | 20º 55’ 03’’ | -98º 15’ 14’’ | 6,10 | Nahualt |
|  |  |  | Ixtaczoquico | 20º 54’ 37’’ | -98º 15’ 54’’ | 5,96 | Nahualt |
| Otomí Valle | Hidalgo | El Cardonal | El Buena | 20º 38’ 02’’ | -99º 08’ 37’’ | 4,06 | Otomi |
|  |  |  | El Deca | 20º 36’ 44’’ | -99º 08’ 27’’ | 5,70 | Otomi |
|  |  |  | San Andrés Daboxthá | 20º 31’ 31’’ | -99º 03’ 50’’ | 5,36 | Otomi |
| Otomí Sierra | Hidalgo | San Bartolo Tutotepec | Santiago | 20º 24’ 00’’ | -98º 13’ 05’’ | 0,24 | Otomi |
|  |  | Tenango de Doria | San Nicolás | 20º 19’ 13’’ | -98º 11’ 16’’ | 28,56 | Otomi |
|  |  |  | Santa Mónica | 20º 18’ 22’’ | -98º 13’ 18’’ | 22,93 | Otomi |
| Maya | Quintana Roo | Felipe Carrillo Puerto | Tihosuco | 20º 12’ 11’’ | -88º 22’ 14’’ | 9,65 | Maya |
|  |  | José María de Morelos | Sabán | 20º 02’ 02’’ | -88º 32’ 19’’ | 9,44 | Maya |

**Table S1. Sample geographic and demographic information. The percentage of indigenous population has been calculated in reference to**

**the municipality (INEGI 2000).**
