# Supplementary material for: Reconstructing the History of Mesoamerican Populations through the Study of the Mitochondrial DNA Control Region
Source: PLoS One. 2012 Sep 19;7(9):e44666. doi: 10.1371/journal.pone.0044666 (PMC3446984; doi:10.1371/journal.pone.0044666)
Supplement: Table S2 — Number of samples (N) and haplogroups found in the eight populations. (DOCX) [file pone.0044666.s007.docx]

**Tabla S2. Number of samples (N) and haplogroups found in the eight populations.**

| Population | N | A2 (%) | B2 (%) | C1 (%) | D1 (%) | H | T | V |
| --- | --- | --- | --- | --- | --- | --- | --- | --- |
| Pima | 49 | 1 (2,04) | 7 (14,28) | 40 (81,63) | 1 (2,04) | 0 | 0 | 0 |
| Mayo | 55 | 13 (23,63) | 17 (30,90) | 7 (12,72) | 18 (32,72) | 0 | 0 | 0 |
| Huichol | 36 | 20 (55,55) | 9 (25,00) | 6 (16,66) | 1 (2,77) | 0 | 0 | 0 |
| Nahua | 192 | 110 (57,29) | 54 (28,12) | 18 (9,37) | 9 (4,69) | 1(0,52) | 0 | 0 |
| Otomí Valle | 81 | 40 (49,38) | 12 (14,81) | 22 (27,16) | 7 (8,64) | 0 | 0 | 0 |
| Otomí Sierra | 94 | 49 (52,12) | 10 (10,63) | 22 (2340) | 10 (10,63) | 1(1,06) | 1(1,06) | 1(1,06) |
| Tepehua | 54 | 34 (62,96) | 14 (25,92) | 3 (5,55) | 2 (3,70) | 1(1,85) | 0 | 0 |
| Maya | 44 | 35 (79,54) | 3 (6,81) | 4 (9,09) | 2 (4,45) | 0 | 0 | 0 |
| Total | 605 | 302 (49,92) | 126 (20,82) | 122 (20,16) | 50 (8,26) | 3 (0,49) | 1 (0,16) | 1 (0,16) |
