# Supplementary material for: Reconstructing the History of Mesoamerican Populations through the Study of the Mitochondrial DNA Control Region
Source: PLoS One. 2012 Sep 19;7(9):e44666. doi: 10.1371/journal.pone.0044666 (PMC3446984; doi:10.1371/journal.pone.0044666)
Supplement: Table S3 — Diversity indices for mtDNA control region (16024-576) sequences and lineages in twelve indigenous populations [15] . N, sample size; K, number of different sequences; S, number of polymorphic sites; Ĥ, sequence diversity; π, nucleotide diversity; θ, mean number of pairwise differences between sequences; D, Tajima test of selective neutrality (s.d. standard deviation). (DOCX) [file pone.0044666.s008.docx]

**Table S3. Diversity indices for mtDNA control region (16024-576) sequences and lineages in twelve indigenous populations** [15]. N, sample size; K, number of different sequences; S, number of polymorphic sites; Ĥ, sequence diversity; π, nucleotide diversity; θ, mean number of pairwise differences between sequences; D, Tajima test of selective neutrality (s.d. standard deviation).

| ***Population*** | ***N*** | ***K*** | ***S*** | ***Ĥ± sd*** | ***π±sd*** | ***θ*** | ***D*** |
| --- | --- | --- | --- | --- | --- | --- | --- |
| **Hualapai** | 54 | 18 | 47 | 0,91±0,021 | 0,0092±0,0003 | 10,57 | 0,08 |
| **Zunis** | 30 | 12 | 40 | 0,88±0,033 | 0,0078±0,0012 | 8,95 | -0,41 |
| **Pimas_k** | 72 | 39 | 61 | 0,97±0,009 | 0,0093±0,0003 | 10,45 | -0,56 |
| **Pápagos** | 38 | 24 | 52 | 0,96±0,021 | 0,0095±0,0008 | 10,84 | -0,44 |
| **Tarahumaras** | 55 | 17 | 44 | 0,91±0,018 | 0,0098±0,0004 | 11,23 | 0,56 |
| **Coras** | 72 | 29 | 56 | 0,95±0,009 | 0,0096±0,0003 | 10,99 | -0,21 |
| **Huichol_k** | 56 | 13 | 41 | 0,88±0,020 | 0,0090±0,0004 | 10,98 | 0,77 |
| **Nahuas At** | 45 | 33 | 76 | 0,98±0,011 | 0,0119±0,0005 | 13,19 | -0,86 |
| **Nahuas Cu** | 29 | 24 | 55 | 0,98±0,012 | 0,0100±0,0009 | 11,38 | -0,70 |
| **Mixtecos** | 65 | 28 | 57 | 0,96±0,009 | 0,0092±0,0006 | 10,43 | -0,44 |
| **Mixes** | 49 | 20 | 48 | 0,93±0,018 | 0,0105±0,0003 | 11,92 | 0,37 |
| **Zapotecos** | 72 | 42 | 80 | 0,98±0,005 | 0,0111±0,0003 | 12,71 | -0,77 |
