# Supplementary material for: Reconstructing the History of Mesoamerican Populations through the Study of the Mitochondrial DNA Control Region
Source: PLoS One. 2012 Sep 19;7(9):e44666. doi: 10.1371/journal.pone.0044666 (PMC3446984; doi:10.1371/journal.pone.0044666)
Supplement: Table S4 — Theta estimators for the twelve populations [15] . (DOCX) [file pone.0044666.s009.docx]

**Table S4. Theta estimators for the twelve populations [15].**

| *Population* | *θ_K_ (95% CI)* | *θ_S_± sd* | *θ_π_± sd* |
| --- | --- | --- | --- |
| Hualapai | 9.09( 5.03-15.91) | 10.53±3.19 | 14.09±7.12 |
| Zunis | 6.91(3.32-14.06) | 10.34±3.53 | 12.34±6.38 |
| Pimas_k | 33.99(21.33-54.18) | 12.79±3.61 | 14.55±7.30 |
| Pápagos | 26.94(14.35-51.24) | 12.37±3.94 | 14.47±7.36 |
| Tarahumaras | 8.03(4.43-14.22) | 9.83±2.99 | 15.57±7.83 |
| Coras | 17.53(10.78-28.23) | 11.55±3.30 | 14.24±7.15 |
| Huichol_k | 5.01( 2.61- 9.26) | 9.14±2.79 | 14.70±7.40 |
| Nahuas_at | 51.27(27.65-97.66) | 17.01±5.14 | 16.72±8.42 |
| Nahuas Cu | 67.91(29.88-165.38) | 15.14±4.98 | 14.24±7.30 |
| Mixtecos | 18.12(10.96-29.70) | 12.22±3.53 | 13.29±6.71 |
| Mixes | 13.10( 7.39-22.93) | 11.60±3.53 | 15.11±7.62 |
| Zapotecos | 41.30(25.91-66.07) | 16.71±4.62 | 16.62±8.29 |
