# Supplementary material for: Reconstructing the History of Mesoamerican Populations through the Study of the Mitochondrial DNA Control Region
Source: PLoS One. 2012 Sep 19;7(9):e44666. doi: 10.1371/journal.pone.0044666 (PMC3446984; doi:10.1371/journal.pone.0044666)
Supplement: Table S5 — AMOVAs based on different classification criteria. We have used the mtDNA control region of twenty populations from Mesoamerica and Aridoamerica. In bold the eight populations studied in this paper. *Significance level 0.05. (DOC) [file pone.0044666.s010.doc]

**Table S5. AMOVAs based on different classification criteria.** We have used the mtDNA control region of twenty populations from Mesoamerica and Aridoamerica. In bold the eight populations studied in this paper. *Significance level 0.05.

| **Grouping criteria** | **Groups** | **Populations** | |  | | **Variance** | | **Indices fixation** | | **P** | |  |
| --- | --- | --- | --- | --- | --- | --- | --- | --- | --- | --- | --- | --- |
| Geography I | North | Zuni, Hualapai, Papago, Pima_k, **Pima**, Tarahumara | | Within populations | | 86,35 | | Fst = 0.13647 | | 0.0000* | |  |
|  | Occident | **Mayo**, Cora, Huichol_k, **Huichol** | | Among populations within groups | | 6,83 | | Fsc = 0.07331 | | 0.0000* | |  |
|  | Oaxaca | Mixteco, Mixe, Zapoteco | | Among groups | | 6.82 | | Fct = 0.06818 | | 0.0009* | |  |
|  | Center | Nahua_at, **Nahuas_Hu**, , Nahua_cu **Otomis_v**, **Otomis_s**, **Tepehua** | |  |  |  |  |  |  |  |  |  |
|  | South | **Maya** | |  |  |  |  |  |  |  |  |  |
| Geography II | North | Zuni, Hualapai, Papago, Pima_k, **Pima**, Tarahumara | | Within populations | | 86.64 | | Fst = 0.13359 | | 0.0000* | |  |
|  | Occident | **Mayo**, Cora, Huichol_k, **Huichol** | | Among populations within groups | | 7.12 | | Fsc = 0.07595 | | 0.0000* | |  |
|  | Center | Nahua_at, **Nahuas_Hu**, , Nahua_cu **Otomis_v, Otomis_s** | | Among groups | | 6,24 | | Fct = 0.06238 | | 0.0009* | |  |
|  | Orient | **Tepehua** | |  |  |  |  |  |  |  |  |  |
|  | Oaxaca | Mixteco, Mixe, Zapoteco | |  | |  | |  | |  | |  |
|  | South | **Mayas** | |  | |  | |  | |  | |  |
| Languages | Isolate | | Zuni | | Within populations | | 86,99 | | Fst = 0,13014 | | 0.0000* | |
|  | Uto-Nahua | | Cora, Papagos, **Pimas**, Pima-k, Tarahumanara, **Huichol**, Huichol_k, **Mayo, Nahuas_Hu**, Nahuas_cu, Nahuas_at | | Among populations within groups | | 11,05 | | Fsc = 0,11267 | | 0.0000* | |
|  | Yumani | | Hualapai | | Among groups | | 1,97 | | Fct = 0,01969 | | 0.2159 | |
|  | Otomangue | | **Otomíes_v**, **Otomíes_v**, Mixtecos, Zapotecos | |  | |  |  |  |  |  |  |
|  | Mixe-Zoquean | | Mixe | |  | |  |  |  |  |  |  |
|  | Totonaca | | **Tepehuas** | |  | |  | |  | |  | |
|  | Maya | | **Mayas** | |  | |  | |  | |  | |
