# Supplementary material for: Reconstructing the History of Mesoamerican Populations through the Study of the Mitochondrial DNA Control Region
Source: PLoS One. 2012 Sep 19;7(9):e44666. doi: 10.1371/journal.pone.0044666 (PMC3446984; doi:10.1371/journal.pone.0044666)
Supplement: Table S6 — FST calculated for twenty populations from Mesoamerica and Aridoamerica. In bold the eight populations studied. (DOC) [file pone.0044666.s011.doc]

**Table S6. F_ST_ calculated for twenty populations from Mesoamerica and Aridoamerica.** In bold the eight populations studied.

|  | Hualapai | Zuni | Pima_k | Papago | **Pima** | Tarahumara | **Mayo** | Cora | **Huichol** | Huichol_k | **Nahua** | **Otomí Valle** | **Otomí Sierra** | **Tepehua** | Nahua_at | Nahua_cu | Mixteco | Mixe | Zapoteco | **Maya** |
| --- | --- | --- | --- | --- | --- | --- | --- | --- | --- | --- | --- | --- | --- | --- | --- | --- | --- | --- | --- | --- |
| Hualapai | 0.0000 |  |  |  |  |  |  |  |  |  |  |  |  |  |  |  |  |  |  |  |
| Zuni | 0.0996 | 0.0000 |  |  |  |  |  |  |  |  |  |  |  |  |  |  |  |  |  |  |
| Pima_k | 0.0558 | 0.0613 | 0.0000 |  |  |  |  |  |  |  |  |  |  |  |  |  |  |  |  |  |
| Papago | 0.0615 | 0.0705 | 0.0079 | 0.0000 |  |  |  |  |  |  |  |  |  |  |  |  |  |  |  |  |
| **Pima** | 0.0964 | 0.1104 | 0.0672 | 0.0714 | 0.0000 |  |  |  |  |  |  |  |  |  |  |  |  |  |  |  |
| Tarahumara | 0.0877 | 0.0936 | 0.0589 | 0.0627 | 0.0873 | 0.0000 |  |  |  |  |  |  |  |  |  |  |  |  |  |  |
| **Mayo** | 0.0655 | 0.0666 | 0.0370 | 0.0391 | 0.0751 | 0.0554 | 0.0000 |  |  |  |  |  |  |  |  |  |  |  |  |  |
| Cora | 0.0681 | 0.0808 | 0.0399 | 0.0430 | 0.0775 | 0.0664 | 0.0448 | 0.0000 |  |  |  |  |  |  |  |  |  |  |  |  |
| **Huichol** | 0.0621 | 0.0752 | 0.0330 | 0.0353 | 0.0720 | 0.0604 | 0.0371 | 0.0276 | 0.0000 |  |  |  |  |  |  |  |  |  |  |  |
| Huichol_k | 0.1008 | 0.0804 | 0.0717 | 0.0761 | 0.1106 | 0.0966 | 0.0715 | 0.0670 | 0.0296 | 0.0000 |  |  |  |  |  |  |  |  |  |  |
| **Nahua** | 0.0493 | 0.0605 | 0.0233 | 0.0260 | 0.0586 | 0.0473 | 0.0266 | 0.0318 | 0.0208 | 0.0591 | 0.0000 |  |  |  |  |  |  |  |  |  |
| **Otomí Valle** | 0.0586 | 0.0709 | 0.0309 | 0.0337 | 0.0664 | 0.0600 | 0.0381 | 0.0395 | 0.0160 | 0.0541 | 0.0178 | 0.0000 |  |  |  |  |  |  |  |  |
| **Otomí Sierra** | 0.0570 | 0.0692 | 0.0293 | 0.0322 | 0.0665 | 0.0572 | 0.0356 | 0.0392 | 0.0323 | 0.0709 | 0.0199 | 0.0234 | 0.0000 |  |  |  |  |  |  |  |
| **Tepehua** | 0.0730 | 0.0861 | 0.0444 | 0.0477 | 0.0820 | 0.0742 | 0.0519 | 0.0527 | 0.0236 | 0.0620 | 0.0325 | 0.0282 | 0.0430 | 0.0000 |  |  |  |  |  |  |
| Nahua_at | 0.0542 | 0.0669 | 0.0230 | 0.0271 | 0.0622 | 0.0553 | 0.0328 | 0.0324 | 0.0269 | 0.0667 | 0.0180 | 0.0231 | 0.0222 | 0.0379 | 0.0000 |  |  |  |  |  |
| Nahua_cu | 0.0506 | 0.0637 | 0.0203 | 0.0240 | 0.0606 | 0.0512 | 0.0287 | 0.0318 | 0.0245 | 0.0655 | 0.0129 | 0.0216 | 0.0198 | 0.0365 | 0.0168 | 0.0000 |  |  |  |  |
| Mixteco | 0.0638 | 0.0764 | 0.0355 | 0.0385 | 0.0686 | 0.0575 | 0.0428 | 0.0457 | 0.0352 | 0.0755 | 0.0290 | 0.0366 | 0.0351 | 0.0503 | 0.0310 | 0.0272 | 0.0000 |  |  |  |
| Mixe | 0.0786 | 0.0919 | 0.0498 | 0.0532 | 0.0884 | 0.0798 | 0.0574 | 0.0529 | 0.0538 | 0.0929 | 0.0428 | 0.0509 | 0.0493 | 0.0650 | 0.0459 | 0.0421 | 0.0554 | 0.0000 |  |  |
| Zapoteco | 0.0526 | 0.0646 | 0.0250 | 0.0274 | 0.0625 | 0.0542 | 0.0318 | 0.0263 | 0.0199 | 0.0603 | 0.0165 | 0.0250 | 0.0238 | 0.0397 | 0.0176 | 0.0164 | 0.0281 | 0.0426 | 0.0000 |  |
| **Maya** | 0.0656 | 0.0766 | 0.0352 | 0.0415 | 0.0781 | 0.0695 | 0.0470 | 0.0498 | 0.0431 | 0.0827 | 0.0217 | 0.0388 | 0.0374 | 0.0522 | 0.0334 | 0.0313 | 0.0454 | 0.0601 | 0.0304 | 0.0000 |
