# Supplementary material for: Reconstructing the History of Mesoamerican Populations through the Study of the Mitochondrial DNA Control Region
Source: PLoS One. 2012 Sep 19;7(9):e44666. doi: 10.1371/journal.pone.0044666 (PMC3446984; doi:10.1371/journal.pone.0044666)
Supplement: Table S7 — Number of haplotypes shared between the twenty populations. Lower hemimatrix shows the number of haplotypes shared in the control region, upper hemimatrix shows the number of haplotypes shared in HV-I region. In bold the eight populations studied in this paper. (DOC) [file pone.0044666.s012.doc]

**Table S7. Number of haplotypes shared between the twenty populations.** Lower hemimatrix shows the number of haplotypes shared in the control region, upper hemimatrix shows the number of haplotypes shared in HV-I region. In bold the eight populations studied in this paper.

|  | Hualapai | Zuni | Pima_k | Papago | **Pima** | Tarahumara | **Mayo** | Cora | **Huichol** | Huichol_k | **Nahua** | **Otomí Valle** | **Otomí Sierra** | **Tepehua** | Nahua_at | Nahua_cu | Mixteco | Mixe | Zapoteco | **Maya** |
| --- | --- | --- | --- | --- | --- | --- | --- | --- | --- | --- | --- | --- | --- | --- | --- | --- | --- | --- | --- | --- |
| Hualapai |  | 3 | 5 | 2 | 1 | 1 | 3 | 2 | 1 | 2 | 3 | 4 | 2 | 2 | 2 | 1 | 2 | 2 | 2 | 2 |
| Zuni | 1 |  | 4 | 2 | 1 | 1 | 3 | 2 | 2 | 3 | 2 | 3 | 2 | 1 | 2 | 2 | 2 | 2 | 2 | 1 |
| Pima_k | 1 | 2 |  | 11 | 3 | 2 | 4 | 1 | 1 | 1 | 3 | 3 | 1 | 1 | 3 | 3 | 1 | 2 | 1 | 2 |
| Papago | 0 | 1 | 16 |  | 3 | 2 | 4 | 2 | 2 | 0 | 4 | 3 | 2 | 2 | 3 | 2 | 1 | 2 | 2 | 2 |
| **Pima** | 0 | 0 | 0 | 0 |  | 2 | 3 | 1 | 1 | 0 | 2 | 2 | 1 | 2 | 3 | 1 | 1 | 1 | 1 | 1 |
| Tarahumara | 0 | 1 | 0 | 0 | 2 |  | 3 | 3 | 1 | 1 | 2 | 1 | 1 | 2 | 1 | 1 | 1 | 1 | 2 | 2 |
| **Mayo** | 0 | 1 | 0 | 1 | 0 | 2 |  | 4 | 3 | 3 | 6 | 3 | 4 | 3 | 3 | 3 | 3 | 3 | 3 | 2 |
| Cora | 0 | 0 | 0 | 0 | 0 | 1 | 1 |  | 5 | 3 | 5 | 4 | 5 | 5 | 4 | 4 | 4 | 3 | 4 | 3 |
| **Huichol** | 0 | 0 | 0 | 1 | 0 | 1 | 2 | 4 |  | 6 | 6 | 4 | 5 | 4 | 4 | 5 | 3 | 2 | 4 | 2 |
| Huichol_k | 0 | 1 | 0 | 0 | 0 | 1 | 1 | 2 | 5 |  | 4 | 4 | 4 | 3 | 3 | 4 | 3 | 2 | 3 | 1 |
| **Nahua** | 1 | 1 | 1 | 0 | 1 | 1 | 2 | 2 | 3 | 2 |  | 13 | 14 | 8 | 8 | 11 | 8 | 6 | 9 | 5 |
| **Otomí Valle** | 1 | 1 | 0 | 0 | 1 | 0 | 0 | 1 | 1 | 1 | 8 |  | 13 | 14 | 8 | 7 | 6 | 5 | 7 | 2 |
| **Otomí Sierra** | 1 | 1 | 0 | 0 | 0 | 1 | 1 | 1 | 1 | 0 | 7 | 7 |  | 7 | 8 | 7 | 6 | 5 | 7 | 3 |
| **Tepehua** | 0 | 0 | 0 | 0 | 1 | 0 | 0 | 1 | 1 | 1 | 5 | 3 | 1 |  | 5 | 3 | 4 | 3 | 4 | 3 |
| Nahua_at | 0 | 0 | 2 | 2 | 1 | 0 | 0 | 1 | 1 | 1 | 3 | 2 | 3 | 3 |  | 6 | 4 | 6 | 5 | 2 |
| Nahua_cu | 0 | 0 | 1 | 0 | 0 | 1 | 0 | 0 | 0 | 0 | 4 | 1 | 1 | 0 | 0 |  | 4 | 4 | 3 | 1 |
| Mixteco | 0 | 0 | 0 | 0 | 1 | 1 | 0 | 0 | 1 | 1 | 0 | 0 | 0 | 0 | 1 | 0 |  | 5 | 4 | 2 |
| Mixe | 0 | 0 | 0 | 0 | 0 | 0 | 0 | 1 | 0 | 0 | 0 | 0 | 0 | 0 | 0 | 0 | 1 |  | 4 | 2 |
| Zapoteco | 1 | 1 | 0 | 1 | 0 | 0 | 1 | 2 | 3 | 1 | 2 | 2 | 2 | 0 | 3 | 0 | 1 | 1 |  | 4 |
| **Maya** | 1 | 1 | 1 | 1 | 0 | 0 | 0 | 0 | 0 | 0 | 2 | 1 | 1 | 1 | 2 | 0 | 0 | 0 | 1 |  |
