# Supplementary material for: Reconstructing the History of Mesoamerican Populations through the Study of the Mitochondrial DNA Control Region
Source: PLoS One. 2012 Sep 19;7(9):e44666. doi: 10.1371/journal.pone.0044666 (PMC3446984; doi:10.1371/journal.pone.0044666)
Supplement: Table S8 — Pearson correlation coefficients (r) and their significance (p) between geographic distance from Bering Strait to the communities and genetic diversity indices. K, number of different sequences; S, number of polymorphic sites; Ĥ, sequence diversity; π, nucleotide diversity; θ, mean number of pairwise differences between sequences. (DOC) [file pone.0044666.s013.doc]

**Table S8.** **Pearson correlation coefficients (r) and their significance (p) between geographic distance from Bering Strait to the communities and genetic diversity indices.** K, number of different sequences; S, number of polymorphic sites; Ĥ, sequence diversity; π, nucleotide diversity; θ, mean number of pairwise differences between sequences.

|  | **r** | **p** |
| --- | --- | --- |
| **K** | 0,082 | 0,492 |
| **S** | 0,122 | 0,397 |
| **Ĥ** | 0,354 | 0,120 |
| **π** | 0,141 | 0,359 |
| **θ** | 0,087 | 0,478 |
